# Investigation of the kinetics of spontaneous combustion of the major coal seam in Dahuangshan mining area of the Southern Junggar coalfield, Xinjiang, China

**DOI:** 10.1038/s41598-020-79223-z

**Published:** 2021-01-13

**Authors:** Li Shen, Qiang Zeng

**Affiliations:** 1 School of Resource and Environment Sciences, Xinjiang University, Urumqi, 830046 China; 2 Institute for Arid Ecology and Environment, Xinjiang University, Urumqi, 830046 China; 3 Key Laboratory of Oasis Ecology of Ministry of Education, Urumqi, 830046 China

**Keywords:** Natural hazards, Engineering

## Abstract

In the present paper, with using diverse methods (including the SEM, the XRD, the TPO, the FTIR, and the TGA) , the authors analysed samples of the major coal seam in Dahuangshan Mining area with different particle sizes and with different heated temperatures (from 50 to 800 °C at regular intervals of 50 °C). The results from SEM and XRD showed that high temperature and high number of pores, fissures, and hierarchical structures in the coal samples could facilitate oxidation reactions and spontaneous combustion. A higher degree of graphitization and much greater number of aromatic microcrystalline structures facilitated spontaneous combustion. The results from TPO showed that the oxygen consumption rate of the coal samples increased exponentially with increasing temperature. The generation rates of different gases indicated that temperatures of 90 °C or 130 °C could accelerate coal oxidation. With increasing temperature, the coal oxidation rate increased, and the release of gaseous products was accelerated. The FTIR results showed that the amount of hydroxide radicals and oxygen-containing functional groups increased with the decline in particle size, indicating that a smaller particle size may facilitate the oxidation reaction and spontaneous combustion of coal. The absorbance and the functional group areas at different particle sizes were consistent with those of the heated coal samples, which decreased as the temperature rose. The results from TGA showed that the characteristic temperature T_3_ declined with decreasing particle size. After the sample with 0.15–0.18 mm particle size was heated, its carbon content decreased, and its mineral content increased, inhibiting coal oxidation. This result also shows that the activation energy of the heated samples tended to increase at the stage of high-temperature combustion with increasing heating temperature.

## Introduction

Coal spontaneous combustion (CSC) is a potential disastrous event related to coal mining. It is known that the coal is rich in the Xinjiang region of China, which has experienced severe coal spontaneous combustions events. These events not only pose a serious threat to the safe production of coal mines but also lead impacts on the regional ecological environment^1,2^. The occurrence of CSC is results from the combined action of internal and external factors. The evolution patterns of the coal oxidation microstructure under different temperatures and oxygen conditions have been compared and analysed by X-ray diffraction (XRD) and Fourier transform infrared (FTIR) spectroscopy^3^. Wang et al. studied this issue from a micro-perspective, relying on quantum chemistry theory and infrared spectroscopy: they investigated the molecular structure of coal and several oxidation mechanisms^4^. Researchers have analysed the microstructures and functional group reactions and examined the change in the main oxygen-containing functional groups during a low-temperature coal oxidation process^5–7^.Nimaje and Tripathy investigated the spontaneous combustion characteristics of Indian coal samples^8^, while Avila et al. studied the effect of coal rock composition on coal spontaneous combustion^9^. Bhoi et al. simulated and investigated the combustion and pyrolysis process of brown coal^10^. Zhang et al. used infrared spectroscopy to monitor the methyl and methylene molecular groups, and their reaction kinetic parameters, during a low-temperature oxidation of different coal grades^11^. Zhang et al. conducted a temperature-programmed oxidation (TPO) experiment to analyse the rate of coal consumption and the intensity of heat release from three coal samples^12^. Using thermogravimetry (TG), Krzysztof et al. investigated the pyrolysis behaviour of 11 solid fuels of 11 different grades to ascertain the relationship between the activation energy and pre-exponential factor^13^. Qi et al. tested the thermodynamic properties associated with the reaction process occurring at low oxygen concentrations and analysed the corresponding kinetic factors^14^. Bai et al. investigated the effects of ionic liquids (ILs) on coal macrostructures, and characteristic parameters of coal samples during low-temperature oxidation were observed and evaluated by FTIR and TG-DSC^15,16^. These studies mainly focused on the effects of internal and external factors (e.g., the coal microstructures, the volatile components, the coal rock components, the indicator gases, the functional groups, and the ambient temperature) on the spontaneous combustion of coal, but few studies have focused on spontaneous combustion in major coal seams in the Xinjiang region, a region that contains more than 40 percent of the coal resources in China and still poses a serious risk of coal fire induced by CSC.

Considering the severe risk of CSC in this region, the authors attempted to investigate the thermodynamic characteristics of the major coal seam in the Dahuangshan mining area using diverse methods to reveal the macro- and micro-scale change characteristics of coal oxidation processes and provide a basis for the prevention and control of CSC in the Dahuangshan mining area.

**Figure 1:**
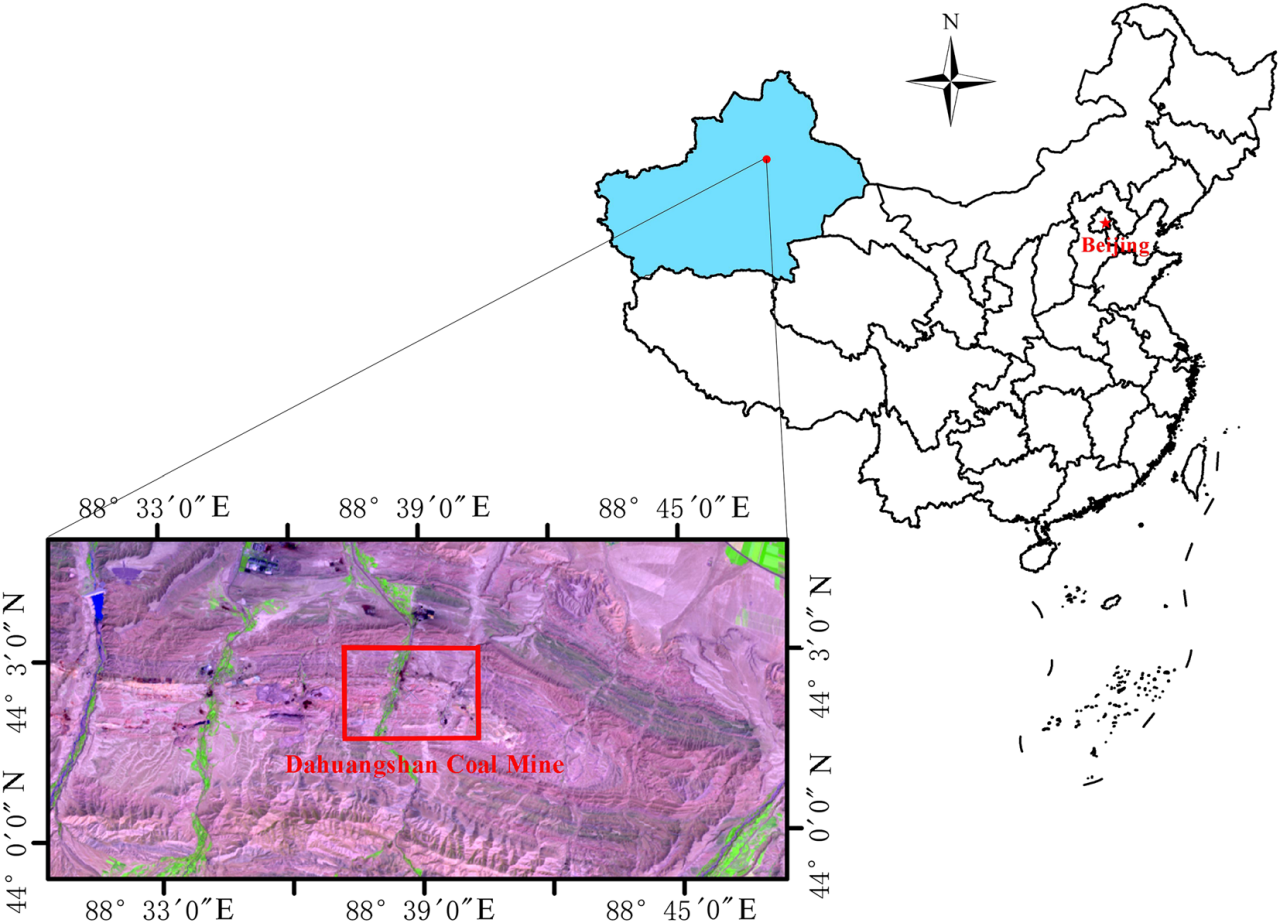
Location of the study area.

The Dahuangshan Mining area is situated in east of the southern Junggar coalfield (see Fig. 1). The major mineable coal seam is A_5,_ with a thickness of approximately 30 m. According to the mining experience of this coal seam, residual coal in gob has been found to be very prone to spontaneous combustion. In fact, coal fires in the Dahuangshan Mining area were extinguished in the mid-1990s with continuous 4 years efforts of extinction. In recent years, with the increase of mining activities in this coal mining area, there is still a risk of CSC in this mining area.

## Materials and methods

### Coal Samples

Collected raw coal was crushed and grilled into samples with particle sizes of 0.25–0.38 mm, 0.15–0.18 mm, 0.109–0.12 mm, 0.08–0.096 mm, and < 0.075 mm. Some of the samples with particle sizes of 0.15–0.18 mm was heated in a muffle furnace at the temperatures of 50 °C, 100 °C, 150 °C, 200 °C, 250 °C, 300 °C, 350 °C, 400 °C, 450 °C, 500 °C, 550 °C, 600 °C, 650 °C, 700 °C, 750 °C, and 800 °C. Table 1 shows the proximate and ultimate values of the coal. The ash was also analysed, as showed in Table 2.

**Table 1:** Proximate and ultimate values for the A_5_ coal seam.

M_ad (_%)	A_ad (_%)	V_ad (_%)	FC_ad (_%)	C_ad (_%)	H_ad (_%)	O_ad (_%)	N_ad (_%)	S_t,__ad (_%)	M_t (_%)
1.74	10.30	38.24	49.72	71.51	4.82	10.35	1.04	0.24	2.6

**Table 2:** Oxides in the ash residue of the coal sample.

SiO_2_ (%)	Al_2_O_3_ (%)	TiO_2_ (%)	K_2_O (%)	Na_2_O (%)	MnO_2_ (%)	Fe_2_O_3_ (%)	CaO (%)	MgO (%)	SO_3_ (%)	P_2_O_5_ (%)
46.57	7.55	1.39	0.37	1.34	0.47	17.35	10.33	3.37	2.80	0.01

The ash residue contained SiO_2_, Fe_2_O_3_, CaO, Al_2_O_3_, MgO, SO_3_, TiO_2_, Na_2_O and so on. The oxides in the ash residue of coal sample were divided into acidic and basic oxides. The acidic oxides included SiO_2,_ Al_2_O_3_, TiO_2_, and the basic oxides included Fe_2_O_3_, Na_2_O, CaO, MgO and K_2_O. The amount of SiO_2_ (46.57%) was in the range of 45–60%, and SiO_2_ has a high ash fusibility, which will reduce the coal combustibility.

### Methods

#### Scanning electron microscope analysis

Coal samples were scanned using a Hitachi Su8000 (Japan) high-resolution field emission electron microscope. Changes of microstructure in coal samples (such as pores, fissures and surface morphology) were investigated before and after heating.

#### XRD analysis

A 18-Kw X-ray powder diffractometer (MacScience, Japan) was used for the XRD analysis, which involved the use of Cu–Ka radiation (step = 0.02034°, counting time = 19.2 s per step, voltage = 40 kV, current = 40 mA, and scanning range = 5–80°).

#### Temperature-programmed oxidation (TPO) analysis

The five coal samples with particle sizes of 0.25–0.38 mm, 0.15–0.18 mm, 0.109–0.12 mm, 0.08–0.096 mm, and < 0.075 mm were mixed. Then, the mixed sample was tested on a BPG-907a experiment platform (XUST, China). The TPO analysis was performed at a heating rate of 0.3 °C per min, heating range of 30–170 °C, and an airflow rate of 120 mL per min. After each 10 °C increment, the exit gas was collected and analysed for quantifying composition and concentration. The composition and concentration of gases include O_2_, CO, CO_2_, and C_n_H_m_. When the temperature of the coal sample exceeded that of the furnace chamber, the TPO test was finished.

#### Infrared spectroscopy analysis

A Vertex 70 Fourier infrared spectrometer (Bruker, Germany) was used for this type of analysis. The experimental conditions were as follows: RES = 4.0 cm^−1^, scans = 120, and wavelength = 400–4000 cm^−1^.

#### TGA

A Hitachi STA 7300 thermal analyser (Japan) was used with nitrogen gas. The experimental conditions included a heating rate = 10 °C per min, flow rate = 200 mL per min, and temperature that ranged from ambient room temperature to 1000 °C.

### Data processing

#### Calculation of the microcrystalline structure parameters

Based on the XRD diffraction pattern of coal, the following microcrystalline structure parameters can be accurately calculated using the Bragg equation and the Scherer formula: the distances between the aromatic monolayers (*d*_*002*_ and *d*_*100*_; 10^–1^ nm), the diameter of the aromatic layer (*L*_*a*_*;* 10^–1^ nm), and the average stacking thickness of the aromatic layers (*L*_*c*_; 10^–1^ nm). d_002_ was calculated using the Bragg angle Eq. (1), while *L*_*c*_ and *L*_*a*_ were calculated using the Scherrer equation^17^:

1 $$ d_{{{002}}} { = }\lambda {/2}\sin \theta_{{{002}}} $$

2 $$ L_{\begin{subarray}{l} {\text{c}} \\ \end{subarray} } = k_{{1}} {\uplambda }/(\beta_{{{002}}} {\text{cos}}\theta_{{{002}}} ) $$

3 $$ L_{{\text{a}}} = k_{{2}} {\uplambda }/(\beta_{{{100}}} {\text{cos}}\theta_{{{100}}} ) $$

where *λ* denotes the wavelength of the incident X-ray, (nm), *θ*_*002*_ and *θ*_*100*_ denote the Bragg angles corresponding to Peak 002 and Peak 100, respectively, (°) ; *β*_*002*_ and *β*_*100*_ denote the half widths of Peak 002 and Peak 100, respectively, (rad); and K_1_ and K_2_ are the microcrystalline shape factors of coal, respectively, (*k*_*1*_ = 0.94, *k*_*2*_ = 1.84). The coal interlayer spacing (*d*_*002*_) ranged between the values typical of cellulose and graphite (3.975 × 10^–1^ nm and 3.354 × 10^–1^ nm, respectively). Analogously, the degree of graphitisation, the degree of graphitization (*P*) can be used to represent the stacking structure of aromatic layers in coal and assess the relative contents of stacking structures in the aromatic and fat layers based on the following equation^18^:

4 $$ P = \frac{{d_{c} - d_{m} }}{{d_{c} - d_{g} }} \times 100\% $$

where *P* denotes the degree of graphitization, *d*_*m*_ denotes the distance between the aromatic layers (nm), *d*_*c*_ denotes the distance between the microcrystalline structure layers of graphite (*d*_*c*_ = 3.975 × 10^–1^ nm), and *d*_*g*_ denotes the distance between the microcrystalline structure layers of the fibre bundles (*d*_*g*_ = 3.354 × 10^–1^ nm).

#### Characteristic temperatures

**Figure 2:**
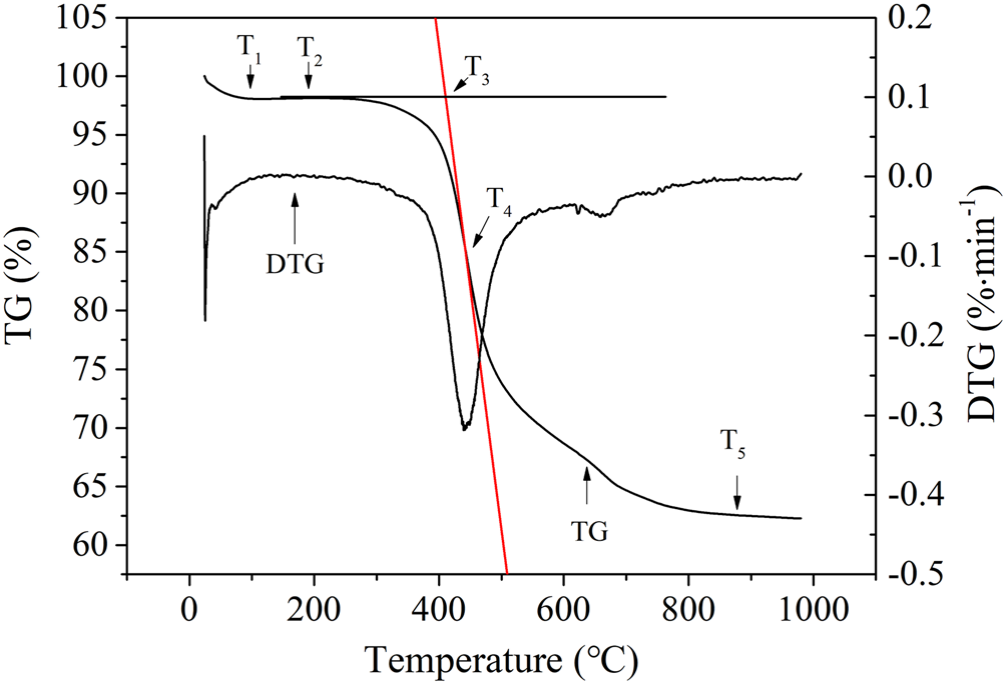
TG curves of the coal samples.

Based on the TGA data, the TG curve were plotted and shown in Fig. 2.

The values of T_1_–T_5_ in Fig. 2 denote the characteristic temperatures of coal at different stages of the TG test: T_1_ denotes the corner point temperature (at which the coal sample gained weight, after water loss and oxygen uptake), T_2_ denotes the initial temperature of pyrolysis, T_3_ denotes the ignition temperature, T_4_ denotes the temperature at which the burning rate was maximized, and T_5_ denotes the burn-out temperature.

#### Calculation of the activation energy

The activation energy was calculated using the Coats–Redfern integral method. Here, the coal–oxygen combustion reaction was viewed as a first-order reaction. According to the Arrhenius law, the reaction rate of coal combustion can be calculated based on the following equation:

5 $$ k = A\exp ( - E/RT) $$

where *k* denotes the coal–oxygen reaction rate constant, *A* denotes the frequency factor, *E* denotes the activation energy (kJ/mol), *T* denotes the reaction temperature (K), and *R* denotes the gas constant (*R* = 8.314 J/(mol⋅K)).

The mass conversion rate during the coal–oxygen reaction process was calculated as follows:

6 $$ \alpha { = }\frac{{m_{0} - m_{t} }}{{m_{0} - m_{\infty } }} $$

where *α* denotes the mass conversion rate during the coal combustion process, *m*_*0*_ is the mass of the coal sample at the start of the TGA experiment, *m*_*t*_ is the mass of the coal sample in the reaction equipment at moment *t* (after the start of the TGA experiment), and *m*_*∞*_ is the mass of the coal sample in the reaction equipment at the end of the TGA experiment. The reaction rate was calculated as follows:

7 $$ \frac{d\alpha }{{dt}} = k(1 - \alpha ) $$

where *k* denotes the chemical reaction rate and *t* is the time.

Then, an integral operation was performed with the Coats–Redfern approximation, yielding the following equations:

8 $$ n = 1,\;\ln \left[ {\frac{ - \ln (1 - \alpha )}{{T^{2} }}} \right] = \ln \left[ {\frac{AR}{{\beta E}}\left( {1 - \frac{2RT}{E}} \right)} \right] - \frac{E}{RT} $$

9 $$ n \ne 1,\;\ln \left[ {\frac{{1 - (1 - \alpha )^{1 - n} }}{{T^{2} (1 - n)}}} \right] = \ln \left[ {\frac{AR}{{\beta E}}\left( {1 - \frac{2RT}{E}} \right)} \right] - \frac{E}{RT} $$

Considering the general reaction temperature range and the activation energy (*E*), the *E/RT* was ≥ 1, and 1–2*RT/E* ~ 1. For n = 1, Eq. (8) could be rewritten as follows:

10 $$ \ln \left[ {\frac{ - \ln (1 - \alpha )}{{T^{2} }}} \right] = - \frac{E}{RT} $$

The above equation was employed to calculate the kinetic parameters of the coal–oxygen reaction in the coal samples. A diagram was generated considering *1n[* − *1n(*1 − *α)/T*^2^*]* for the vertical axis and 1/*T* for the horizontal axis. A linear fitting was then performed on the diagram, and the activation energy was determined according to the slope of the fitting line.

## Results and discussions

### Coal micromorphology

**Figure 3:**
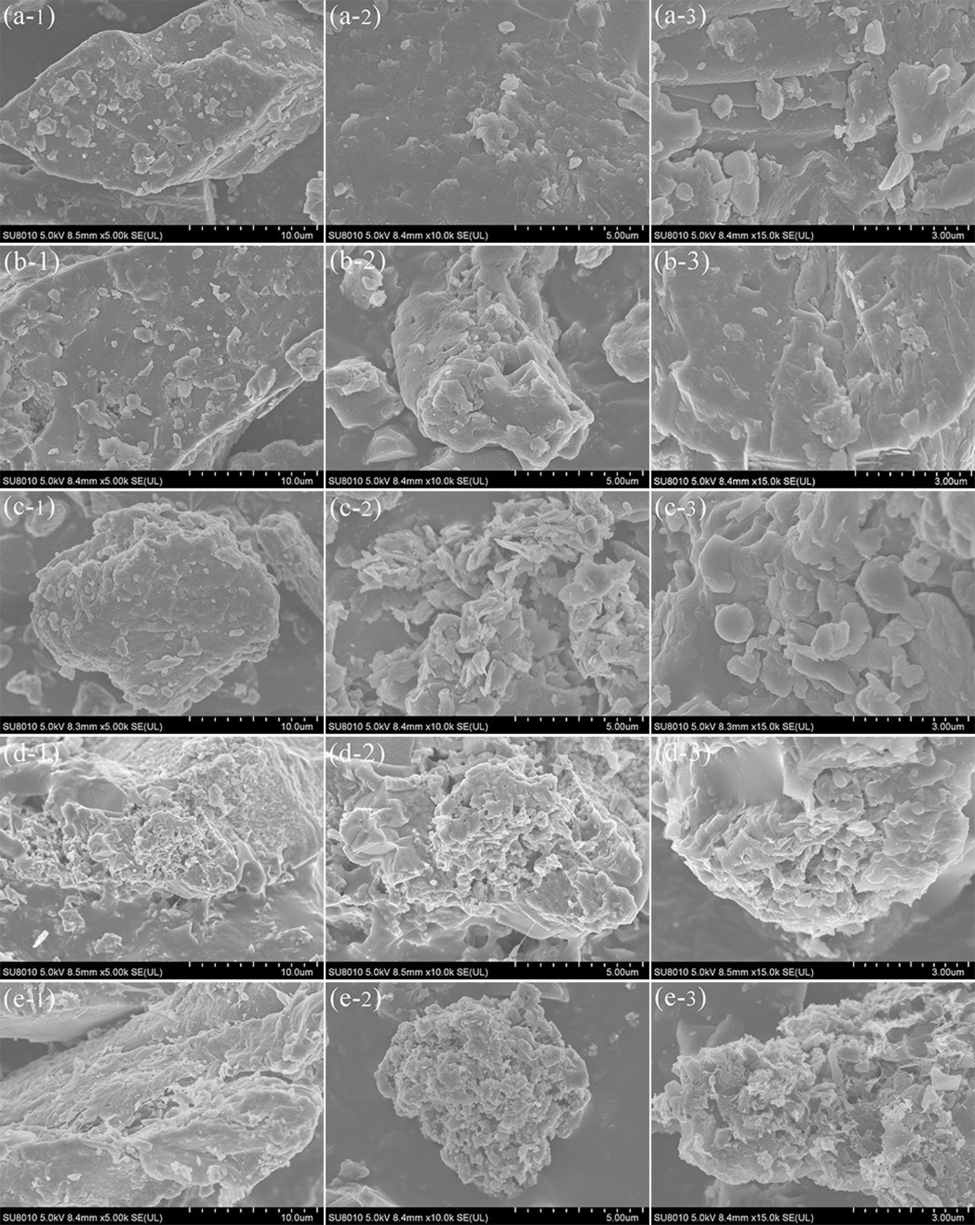
SEM images of the coal sample with particle sizes of 0.15–0.18 mm before and after heating.

The raw coal sample with particle sizes between 0.15 and 0.18 mm was scanned before and after it was heated to 200 °C, 400 °C, 600 °C, and 800 °C. Figure 3 shows the scan images captured at 5000×; 10,000×; and 15,000× magnification.

As shown in Fig. 3, the initial raw coal sample presented a smooth surface and few pores or fissures; moreover, most of the fissures were on the non-stratified surface. After the coal sample was heated to 200 °C, its surface was still non-stratified and presented few pores or fissures; after it was heated to 400 °C, a few stratifications and scraps appeared; after it was heated to 600 °C, its surface became rough, the number of pores and fissures increased, and its specific surface area increased; finally, after it was heated to 800 °C, its surface showed obvious stratification, and many pores and laminations could be observed. Overall, a high heating temperature, a high number of pores and fissures, and a larger superficial area apparently facilitated the extension and connection of microfissures in the coal sample, promoting its oxidation.

### Microcrystalline structure analysis

#### Microcrystalline structures

**Figure 4:**
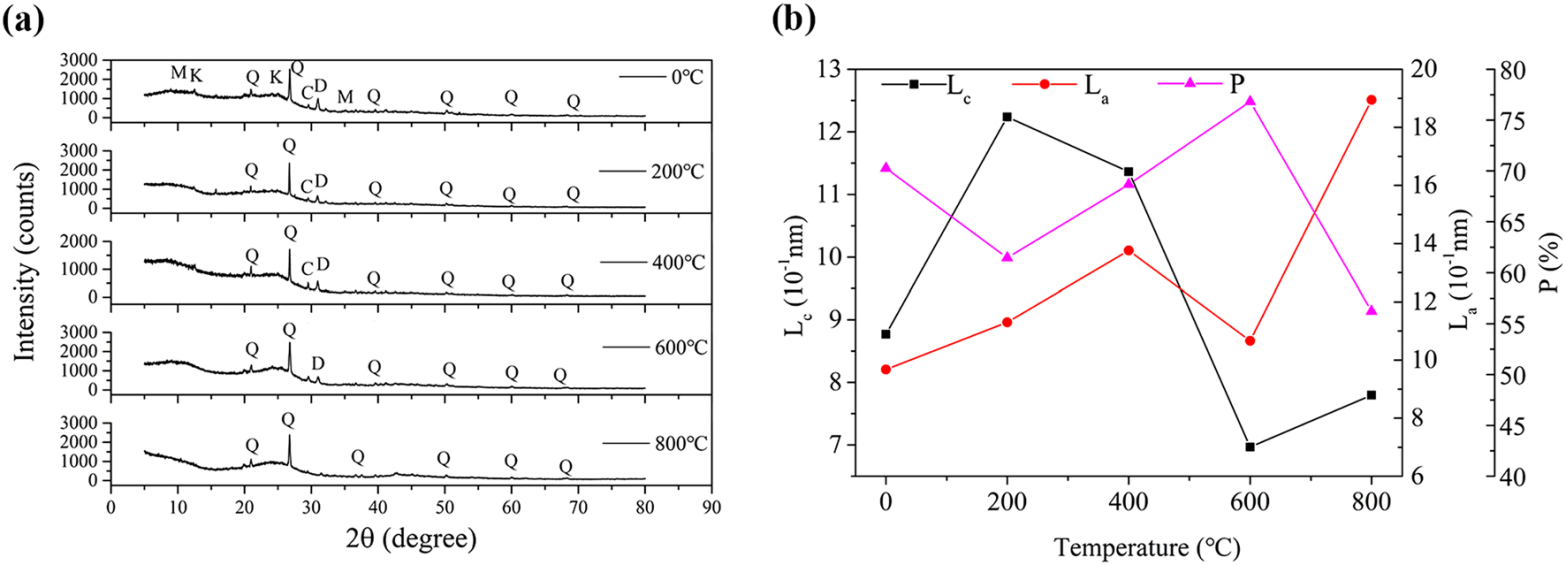
Results from XRD experiments [**a** XRD spectrum at different temperatures; **b** microcrystalline structure parameters (Q–quartz, K– kaolinite, D–dolomite, C–calcite, M–muscovite)].

Coal contains both elementary macromolecular and secondary reticular structures. Reticular structures result from the cross-linking of stacked aromatic nuclei, aliphatic side chains, hydrogen-bonding cations connected through oxygen-containing functional groups, and electric charges. The XRD spectrum of the experimental coal sample with 0.15–0.18 mm particle sizes (Fig. 4a) shows a certain regularity and contains two obvious peaks: Peak 002 (at ~ 25°) and Peak 100 (at ~ 44°). Peak 002 resulted from the superposition of the γ and 002 bands and became narrower and more intense as the coal metamorphic grade increased. Peak 100 reflected the condensation degree of the aromatic nuclei (i.e., the size of the carbon net layer in the aromatic nuclei) and was particularly obvious in the case of high metamorphic grade (i.e., degree of graphitization), suggesting a better directional arrangement of the aromatic layers in the coal molecules.

Through the analysis of the XRD spectrum and related calculation, the microcrystalline structure of the aromatic series (i.e., structural alignment, size, bond length, and atom distribution) could be determined (see Table 3).

**Table 3:** Microcrystalline structure parameters and degree of graphitization of the coal sample with particle sizes between 0.15 and 0.18 mm at different temperatures.

Coal sample (℃)	d_002_ (10^–1^ nm)	L_c_ (10^–1^ nm)	L_a_ (10^–1^ nm)	P (%)
0	3.5387	8.7686	9.6753	70.26
200	3.5934	12.2366	11.2979	61.45
400	3.5485	11.3625	13.7598	68.67
600	3.4980	6.9673	10.6618	76.81
800	3.6258	7.7958	18.9448	56.23

An increase in the heating temperature was accompanied by an increase in the diameter (L_a_) of the aromatic layers. Under the same conditions, the average stacking thickness (L_c_) first increased and then decreased; meanwhile, the graphitization degree first decreased, then increased, and finally decreased (Fig. 4b). Overall, a higher degree of graphitization led to the development of a higher number of aromatic microcrystalline structures.

#### Mineral components

As showed in Fig. 4a, the raw coal sample with 0.15–0.18 mm particle sizes included several mineral components (i.e., quartz, dolomite, kaolinite, calcite, and muscovite). After being heated to 200 °C, the sample contained quartz, kaolinite, calcite and dolomite; after being heated to 400 °C, the sample contained quartz, dolomite, kaolinite and calcite; after being heated to 600 °C, the sample contained quartz, calcite and dolomite; finally, after being heated to 800 °C, the sample contained almost exclusively quartz. These results showed that the variety of mineral components in the coal sample decreased as the heating temperature increased; additionally, quartz was observed under all the tested heating temperatures.

### TPO experimental data

During the adiabatic TPO experiment, the critical temperature of coal samples with different particle sizes and the concentrations of O_2_, CO_n_, and C_n_H_m_ in relation to the reaction temperature during low-temperature oxidation were measured (see Table 4).

**Table 4:** Data obtained from the TPO experiment.

Parameter	Oven temperature (℃)	Coal temperature (℃)	O_2_ (%)	N_2_ (%)	CO (ppm)	CO_2_ (ppm)	CH_4_ (ppm)	C_2_H_6_ (ppm)	C_2_H_4_ (ppm)
	30	30.00	20.86	74	5.57	872	1633	5.661	
	40	40.00	20.51	77.39	3.391	1189	2019	131.4	
	50	50.00	20.25	74.97	4.997	1545	2115	163.4	
	60	60.00	20.14	73.45	16.17	2855	2290	138.9	
	70	70.00	19.76	72.56	38.96	3918	3038	141.8	
	80	80.00	19.19	74.56	83.62	4147	4512	162.6	
	90	90.00	18.58	75.24	176.9	4492	6376	175.2	
	100	100.00	17.65	76.08	377.3	5411	7436	185.9	
	110	110.00	16.7	77.25	682.8	6659	9144	200.4	
	120	120.00	16.05	78.19	1215	7034	10,890	210.1	
	130	130.00	15.82	80.64	1522	8880	12,710	215.6	
	140	140.26	16.6	83.27	2276	11,680	12,480	220.1	
	150	150.00	15.5	74.71	3421	13,672	13,280	215.8	
	160	160.00	12.89	82.94	4752	16,580	14,540	257.4	
	170	170.00	10.11	82.3	5013	19,180	17,450	259.4	56.19

**Figure 5:**
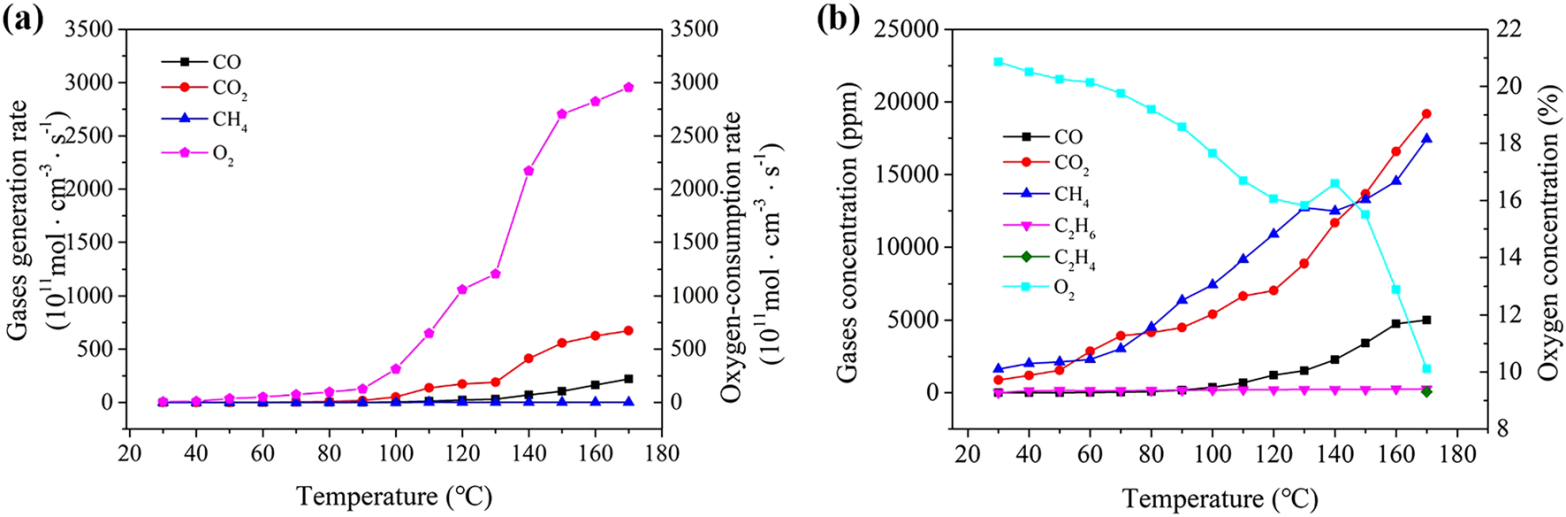
Results from TPO experiments (**a** oxygen consumption and gas generation rates; **b** concentrations of oxygen and gases concentration).

The oxygen consumption rate, an index of coal oxidation, could be calculated based on oxygen concentration. The plot in Fig. 5a shows the relationship between the oxygen consumption rate and temperature: as temperature rose, the oxygen consumption rate of the coal sample increased continuously and exponentially. At low temperature, the interior of the coal body underwent a physicochemical adsorption reaction; as the reaction reached a balanced state and the temperature rose, the coal–oxygen chemical reaction became dominant, leading to the formation of numerous oxygen-consuming functional groups and continuously raising the oxygen consumption rate. Overall, a higher concentration of carbon corresponded to a higher graphitization degree and to a more stable molecular coal structure (lowering the chance of oxidation).

As shown in Fig. 5a, the generation rates of CO and CO_2_ increased as the temperatures increased, while that of CH_4_ varied only slightly under the same conditions. Additionally, the concentrations of CO and CO_2_ increased slowly until the temperature reached 90 °C and then increased rapidly, indicating an acceleration of the oxidation reaction. Subsequently, after the temperature exceeded 130 °C, the concentrations of CO and CO_2_ increased sharply, indicating a further acceleration of the oxidation reaction.

As shown in Fig. 5b, the concentration of O_2_ tended to decrease as the temperature increased; meanwhile, the concentrations of CO, CO_2_, and CH_4_ increased, and that of C_2_H_6_ varied only slightly. Notably, C_2_H_4_ was not detected before the heating temperature reached 170 °C; this gas should have been released only once the heating temperature reached a certain threshold. These results indicate that a temperature rise may boost the low-temperature oxidation rate of coal and accelerate the release of gaseous products. Throughout the whole TPO experiment, no C_2_H_2_ was detected: the generation of C_2_H_2_ likely requires temperatures > 180 °C.

### Functional groups analysis

**Figure 6:**
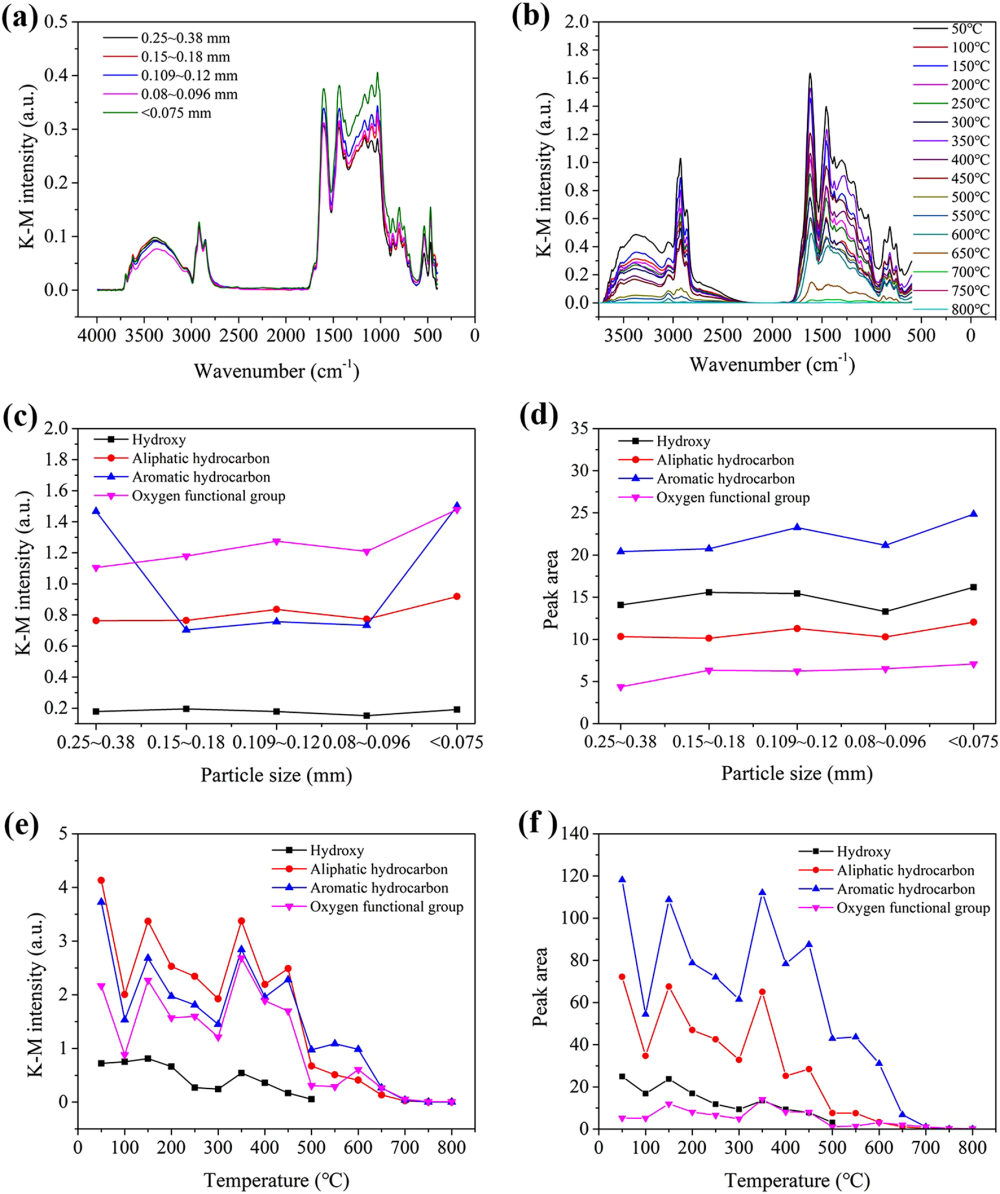
Results from FTIR experiments: (**a** the infrared spectra with different particle sizes; **b** the infrared spectra with heated samples; **c**,**d** the absorbance and peak areas of the functional groups with different particle sizes respectively; **e**,**f** the absorbance and peak areas of functional groups of heated samples respectively).

Table 5 lists the functional groups detected through the in situ infrared analysis of coal samples^19^. Figure 6a,b show the spectra of the samples with different particle sizes and of a sample heated to different temperatures, respectively. Moreover, Fig. 6c,d show the infrared spectral absorbance and the absorption peak areas, respectively, of samples with different particle sizes. Figure 6e,f show the absorbance and absorption peak areas of a sample heated to different temperatures.

**Table 5:** Absorption peak characteristics of the coal samples.

Spectral peak no.	Position of the spectral peak (cm^−1^)	Functional group	Assignment of the spectral peak
1	3697–3625	–OH	Free hydroxyl
2	3624–3613	–OH	Intra-molecular hydrogen bonds
3	3500–3200	–OH	Hydroxyl stretching vibrations of phenols, alcohols, carboxylic acids, peroxides, and water
4	3085–3030	–CH	Aromatics CH stretching vibration
5	2975–2915	–CH_2_, –CH_3_	Benzene ring and aliphatic methyl, methylene antisymmetric stretching vibration
6	2875–2858	–CH_2_, –CH_3_	Methyl symmetrical stretching vibration
7	1625–1575	C=C	Aromatic ring C=C stretching vibration
8	1470–1430	–CH_2_–CH_3_	Methyl antisymmetric stretching vibration
9	1379–1373	–CH_3_	Methylene shear vibration
10	1350–1130	C–O	Phenol, alcohol, ether, and ester oxygen bond
11	1100–1000	Si–O	Si–O ether bond
12	900–700	–CH	Outer bending vibration of various substituted
13	540	–S–S–	Characteristic peak of disulphide bond
14	475	–SH	SH absorption peak of organic sulphur

The coal samples with different particle sizes shared the same variety of functional groups; however, the concentrations of these functional groups were different and followed a certain trend (Fig. 6a).Wavenumbers of 3697–3625 cm^−1^, 3624–3613 cm^−1^, and 3500–3200 cm^−1^ corresponded to the OH (i.e., free hydroxide), –OH (i.e., intramolecular hydrogen bonds) and –OH (i.e., hydroxyl stretching vibrations of phenols, alcohols, carboxylic acids, peroxides, and water) functional groups, respectively. The coal sample with particle sizes of 0.15–0.18 mm showed the highest infrared absorption peak, followed by the samples with particle sizes of < 0.075 mm, 0.25–0.38 mm, 0.109–0.12 mm, and 0.08–0.096 mm. Wavenumbers of 3085–3030 cm^−1^ corresponded to the –CH (aromatics CH stretching vibration) functional group, while those of 2975–2915 cm^−1^ were associated with –CH_2_ and –CH_3_ groups (benzene ring and aliphatic methyl, methylene antisymmetric stretching vibration), and those of 2875–2858 cm^−1^ were associated with –CH_2_ and –CH_3_ groups(methyl symmetrical stretching vibration). Overall, the absorption peaks of methyl and methylene were stronger in the coal samples with particle sizes of < 0.075 mm or 0.109–0.12 mm, and were weaker in those with particle sizes of 0.08–0.096 mm. Wavenumbers of 1625–1575 cm^−1^ were associated with the functional group C=C (aromatic ring C=C stretching vibration). The highest absorption peaks were observed for the coal samples with particle sizes < 0.075 mm, while the lowest were observed for those with particle sizes of 0.25–0.38 mm. Wavenumbers of 1449–1439 cm^−1^ were associated with the functional group –CH_2_–CH_3_ (methyl antisymmetric stretching vibration), those of 1379–1373 cm^−1^ with –CH_3_ groups (methylene shear vibration), and those of 1350–1130 cm^−1^ with C–O groups (phenol, alcohol, ether, and ester oxygen bond). The concentrations of methyl and methylene generally increased with the particle size. The coal sample with particle sizes < 0.075 mm showed the highest methyl and methylene infrared absorption peaks, followed by the coal samples with particle sizes of 0.109–0.12 mm, 0.08–0.096 mm, 0.15–0.18 mm, and 0.25–0.38 mm. Wavenumbers of 900–700 cm^−1^ were associated with the functional group –CH (i.e., outer bending vibration of various substituted aromatic hydrocarbons). A decrease in the coal sample particle size resulted in an increasingly distinct infrared absorption spectrum and to increasingly stronger absorption peaks. Wavenumbers of 540 cm^−1^ and 475 cm^−1^ were associated with the functional groups –S–S– (i.e., with the characteristic peak of disulphide bond) and –SH (i.e., with the SH absorption peak of organic sulphur), respectively. A decline in particle size corresponded first to an increase, then to a decrease, and finally to another increase of the absorption peak intensity.

As shown in Fig. 6b, hydroxide radicals were present in the raw coal samples, as well as in those heated to temperatures of 50–500 °C. At temperatures of 500–800 °C, the absorption peak of the hydroxide radicals could be ignored, while those of the –OH functional groups, free hydroxyl groups, and intramolecular hydrogen bonds decreased with rising temperature. The absorption peaks corresponding to the hydroxyl stretching vibrations of phenols, alcohols, carboxylic acids, peroxides, and water decreased at temperatures from 50 to 100 °C, increased from 100 to 150 °C, decreased from 150 to 300 °C, increased from 300 to 350 °C, and finally decreased from 350 to 500 °C. The absorption peak corresponding to the aromatics CH stretching vibration decreased from 50 to 100 °C, increased from 100 to 150 °C, decreased from 150 to 300 °C, increased from 300 to 350 °C, decreased from 350 to 400 °C, increased from 400 to 450 °C, and finally decreased from 450 to 800 °C. The absorption peaks corresponding to the benzene ring and aliphatic methyl, methylene antisymmetric stretching vibration and methyl symmetrical stretching vibration of –CH_2_ and –CH_3_ (at 2920 cm^−1^ and 2850 cm^−1^, respectively) decreased from 50 to 100 °C, increased from 100 to 150 °C, decreased from 150 to 300 °C, increased from 300 to 350 °C, decreased from 350 to 400 °C, increased from 400 to 450 °C, and finally decreased from 450 to 550 °C. At temperatures of 550 to 800 °C, the functional group could be ignored; moreover, methyl and methylene facilitated the composite reaction of oxygen (a higher absorbance of these compounds enhanced the spontaneous combustion oxidation of coal). The aromatic ring C=C stretching vibration corresponded to 1604 cm^−1^, that of –CH_2_–CH_3_ (i.e., methyl antisymmetric stretching vibration) to 1449–1439 cm^−1^, that of –CH_3_ (i.e., methylene shear vibration) to 1379–1373 cm^−1^, that of C–O (i.e., phenol, alcohol, ether, and ester oxygen bond) to 1350–1130 cm^−1^, and those of Si–O, Si–O–Si, and Si–O–C (i.e., Si–O ether bond) to 1100–1000 cm^−1^. The intensities of their respective absorption peaks increased as followed: 50 °C > 350 °C > 150 °C > 450 °C > 400 °C > 200 °C > 250 °C > 100/300 °C > 500/550 °C > 600 °C > 650 °C. At temperatures of 650–800 °C, these functional groups could be ignored, and the peak corresponding to C=C was overlapped with those corresponding to the other functional groups: the intensity of the C=C spectral peak increased significantly. As the coal metamorphic grade (i.e., the temperature stability) increased, the intensity of the above spectral peaks gradually declined. The outer bending vibration of various substituted aromatic hydrocarbons corresponded to the interval 900–700 cm^−1^. The corresponding absorption peaks tended to decrease with rising temperature; in addition, this functional group could be ignored at temperatures between 700 and 800 °C.

As shown in Fig. 6c,d, the absorbance of the functional groups in the coal samples tended to increase with the particle size, while the concentration of aromatic hydrocarbons first decreased sharply and then increased. Moreover, the peak area of the functional groups increased with the particle size, and the maximum concentration of functional groups was found in the coal sample with particle sizes < 0.075 mm. The concentrations of hydroxide radicals and oxygen-containing functional groups increased with the decline in particle size, indicating that the presence of small particles enhanced the oxidation and spontaneous combustion of coal.

As shown in Fig. 6e,f, the absorbance and peak areas of the functional groups in the coal sample heated to different temperatures followed the same trend: they both decreased with rising temperature. Their concentrations followed this order: aromatic hydrocarbons > aliphatic hydrocarbons > hydroxide radicals > oxygen-containing functional groups. Hydroxide radicals and oxygen-containing functional groups are important indicators of coal spontaneous combustion. When the heating temperature was > 500 °C, the hydroxide radicals and the oxygen-containing functional groups participated in the oxidation reaction. Compared to that in the unheated coal samples, the reactivity of the functional groups in the heated coal samples was reduced, and these groups did not enhance the oxidation reaction.

### TG analysis

**Figure 7:**
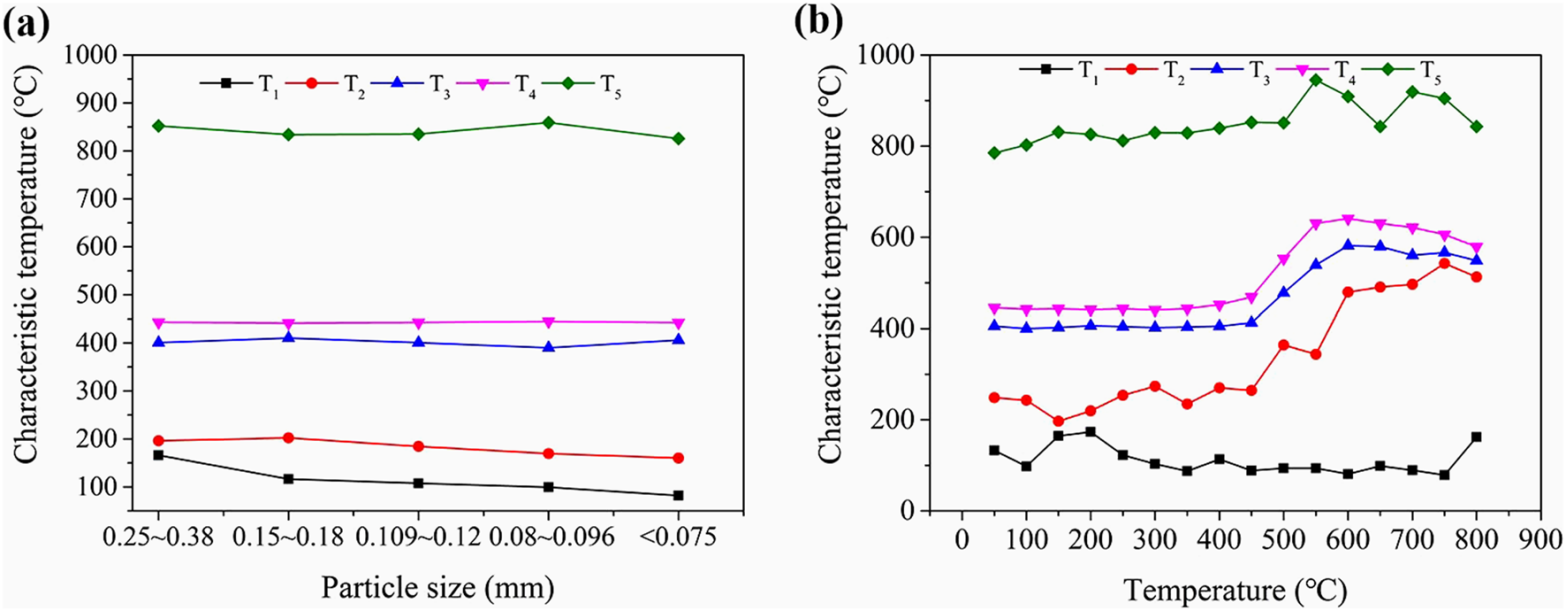
Results from TG experiments (**a** the characteristic temperatures with different particle sizes; **b** the characteristic temperatures of heated samples).

Based on the TG data, the characteristic temperatures (T_1_, T_2_, T_3_, T_4_, and T_5_) were determined. Figure 7a,b show the variation in these temperatures depending on the coal sample particle sizes and heating temperatures.

As shown in Fig. 7a, a decrease in particle size did not translate into a significant variation of T_3_ or T_4_, while T_1_, T_2_, and T_5_ declined; the highest characteristic temperatures were observed for the samples with the largest particle sizes. T_3_ declined with the decrease in particle size: lower particle sizes corresponded to lower ignition temperatures (i.e., the coal sample was more likely to combust spontaneously). As shown in Fig. 7b, T_1_ fluctuated as the temperature increased, while T_2_, T_3_, T_4_, and T_5_ showed an overall increase; meanwhile, the coal content per unit mass decreased and the mineral content per unit mass increased, impeding the coal oxidation reaction.

**Figure 8:**
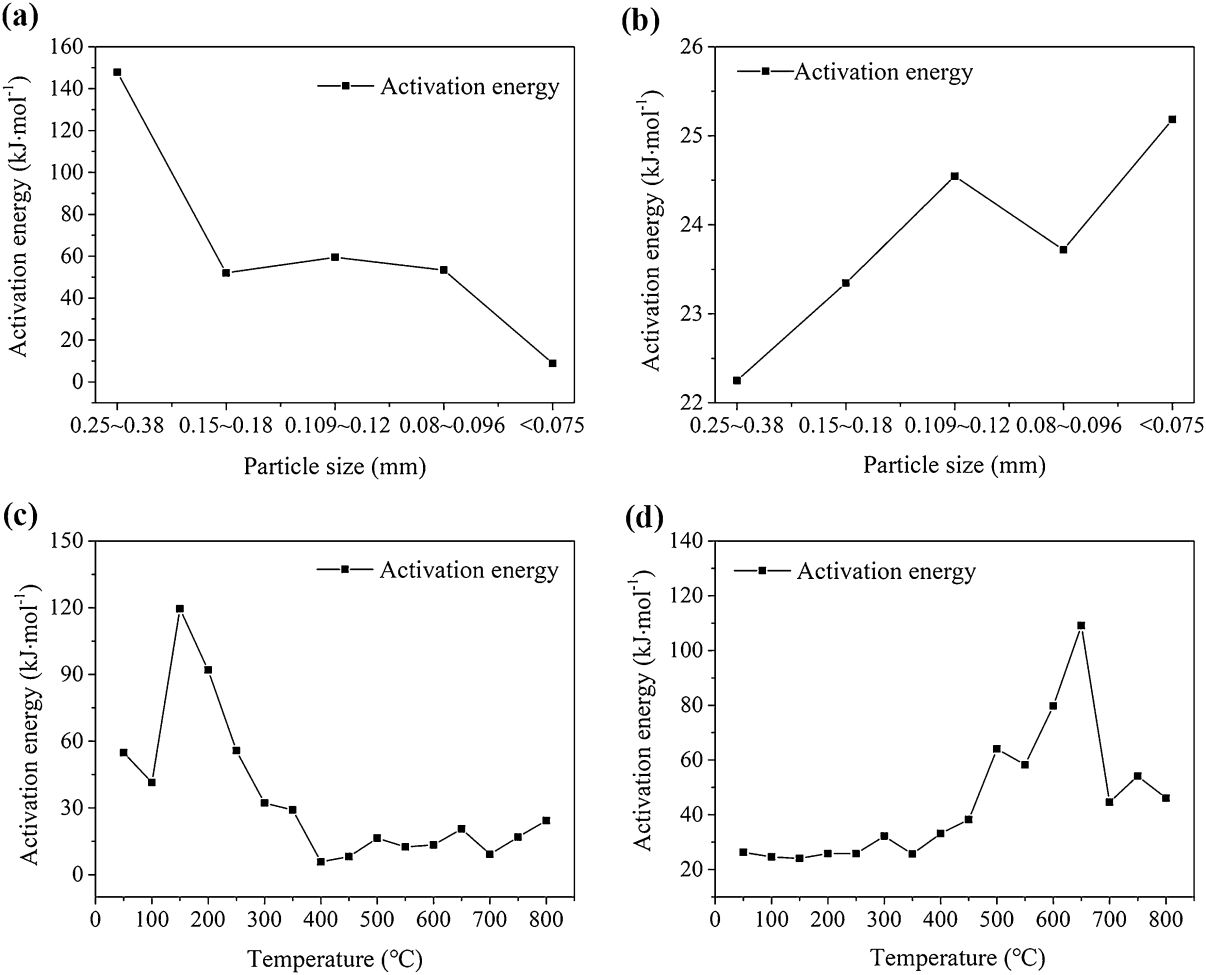
Results from TG experiments (**a**,**b** the activation energy with different particle sizes in stages T_1_–T_2_ and T_3_–T_5_ respectively; **c**,**d** the activation energy of heated samples in stages T_1_–T_2_ and T_3_–T_5_ respectively).

Based on the TG data, the activation energies for the stages of T_1_–T_2_ and T_3_–T_5_ were calculated using the Coats–Redfern integral method. Figure 8 shows their variation.

As shown in Fig. 8a, the activation energies of the coal samples in the T_1_–T_2_ stage generally decreased with the particle size. The activation energies of the coal samples with different particle sizes varied in the following order: coal sample with particle sizes of 0.25–0.38 mm > coal sample with particle sizes of 0.109–0.12 mm > coal sample particle sizes of 0.08–0.096 mm > coal sample particle sizes of 0.15–0.18 mm > coal sample with particle sizes < 0.075 mm. The activation energies of the coal samples in stage T_3_–T_5_ increased with decreasing particle size (Fig. 8b). The activation energies of the coal samples with different particle sizes varied as follows: coal sample with particle sizes < 0.075 mm > coal sample with particle sizes of 0.109–0.12 mm > coal sample with particle sizes of 0.08–0.096 mm > coal sample with particle sizes of 0.15–0.18 mm > coal sample with particle sizes of 0.25–0.38 mm. These results suggest that both lower coal activation energy and higher infrared absorption peaks facilitated the coal–oxygen reaction of the samples.

The reaction activation energy (in stage T_1_–T_2_) of the coal sample heated to different temperatures first increased, then decreased, and finally increased (Fig. 8c,d): at the stage of low-temperature oxidation, the coal sample was more likely to combust spontaneously under rising temperature. The reaction activation energy in stage T_3_–T_5_ first increased and then decreased, but it showed a general increasing trend. Finally, at temperatures from 450 to 800 °C, the reaction activation energy increased. One of the primary reasons for this increase could be that after the coal sample was heated to a certain temperature, the carbon content decreased and the ash content increased moderately, reducing the overall reactivity of the coal sample.

## Conclusions

Based on the above analysis of coal samples with different particle sizes and of a coal sample heated to different temperatures, the following conclusions were obtained:

1. The pores and roughness of coal samples increased with the heating temperature; meanwhile, the degree of graphitization decreased, then increased, and finally decreased again. Higher degrees of graphitization and the development of aromatic microcrystalline structures facilitated the spontaneous combustion of coal.
2. Under rising temperature, the oxygen consumption rate of coal samples being tested increased continuously and exponentially, while the low-temperature oxidation rate of the coal increased, and the release of gaseous products was accelerated. Based on the generating rates of different gases, it can be inferred that temperatures of 90 °C and 130 °C accelerated the oxidation reaction.
3. The amount of hydroxide radicals and oxygen-containing functional groups increased with decreasing particle size: smaller particle sizes likely facilitated the oxidation and the spontaneous combustion of coal. After the coal sample was heated to different temperatures, the absorbance and peak areas of the functional groups followed the same trend: they both decreased under rising temperature. Hydroxide radicals and oxygen-containing functional groups were important indicators of coal spontaneous combustion.
4. With decreasing particle size, the characteristic temperatureT_4_ varied slightly, while T_1_, T_2_, T_3_, and T_5_ all tended to decrease. In particular, T_3_ decreased with the particle size: the smaller the particle size, the lower was the ignition temperature of the coal sample. Additionally, with increasing temperature, the carbon content of coal samples decreased, and their mineral content increased, inhibiting the coal oxidation reaction. As the heating temperatures increased, the activation energies of the coal samples tended to increase at the stages of low-temperature oxidation and high-temperature combustion.
